# RECOVER-E – a mobile app for patients undergoing total knee or hip replacement: study protocol

**DOI:** 10.1186/s12891-020-3090-2

**Published:** 2020-02-04

**Authors:** Anja Stauber, Nadine Schüßler, Sarah Palmdorf, Nina Schürholz, David Bruns, Jürgen Osterbrink, Nadja Nestler

**Affiliations:** 1 Institute of Nursing Science and Practice, Paracelsus Medical University, Strubergasse 21, 5020 Salzburg, Austria; 2 Faculty of Health Sciences, Bielefeld University, Universitätsstraße 25, 33615 Bielefeld, Germany

**Keywords:** mHealth, eHealth, Osteoarthritis, Hip, Hip replacement, THR, Knee, Knee replacement, TKR, Joint replacement, Education

## Abstract

**Background:**

Total knee replacement (TKR) or total hip replacement (THR) are common and effective procedures in patients with osteoarthritis (OA) to restore physical function and reduce joint related pain. Patient education plays an important role in the treatment process aiming to develop necessary self-management skills to facilitate recovery and ensure long-term success. We have developed a mobile app (RECOVER-E) for iOS and Android smartphones which provides important information on the preoperative phase, surgery and recovery. The concomitant study will determine the efficacy of RECOVER-E on patients’ surgical outcomes.

**Methods/design:**

This study is a non-randomized, multi-centre (4 sites), double-arm, controlled trial with 1:1 assignment. 160 patients undergoing primary TKR or THR will be recruited from January until October 2019 in 4 German hospitals. Both groups will receive standard care. Additionally, the intervention group will use the app RECOVER-E. Measurements will be taken 4–6 weeks before surgery, on the day of admission to the hospital, on the first and 7th postoperative day and 3 months post-surgery. Primary outcome will be self-reported physical function measured on the activities of daily living (ADL) subscale of the Knee injury and Osteoarthritis Outcome Score (KOOS) and the Hip disability and Osteoarthritis Outcome Score (HOOS) for patients with knee and hip osteoarthritis, respectively.

Secondary outcomes include the subscales for pain, symptoms, function in sport and recreation and knee/hip-related quality of life of the HOOS and KOOS, preoperative anxiety, measured by the Hospital Anxiety and Depression Scale (HADS), as well as, pain at rest and pain during activity measured by a numerical rating scale (NRS). Primary endpoint is 3 months post-surgery.

**Discussion:**

Mobile Health (mHealth) has become increasingly important in patient-centred health care aiming to enhance patient involvement and self-management capabilities. To our knowledge this is the first study to investigate the effect of an evidence-based mobile app on patient reported outcomes after joint replacement. This study should provide evidence supporting the use of mHealth to facilitate recovery and open up new possibilities for patient care in joint replacement.

**Trial registration:**

DRKS Data Management retrospectively registered. DRKS-ID: DRKS00012744.

## Background

Osteoarthritis (OA) is worldwide one of the most common joint diseases that causes pain and functional disability and results in an increasing number of total hip or knee replacement procedures [1–4]. In Germany 162,524 hip replacement procedures and 178,479 knee replacement procedures were performed in 2016 due to hip or knee OA, respectively [5]. Due to the ageing of the population and an increase in obesity, the incidence and prevalence of these inflammatory joint diseases is growing and expectations to maintain a physically active lifestyle will most likely lead to a higher demand for surgery at a younger age [6, 7]. Thus, the requirement for total joint replacement is expected to increase steadily [6–8]. The main objective of joint replacement procedures is to restore greatest possible physical function and reduce pain. Especially in the elderly, these factors are considered as having an important impact on self-determination and social participation, and therefore, on quality of life [3, 9].

Total hip replacement (THR) and total knee replacement (TKR) are generally successful and lead to good clinical outcomes, but the procedure can be physically and psychologically challenging for patients [10, 11]. Same-day admissions in modern hospital practices and reduced length of hospital stay do not leave much time for patients to adjust to their situation [12]. Many people fail to recover optimally and continue to experience pain and functional problems [2, 10].

Preoperative education appears to have a positive effect on the patients’ postoperative coping abilities [13]. Pain disability, in particular, is largely influenced by how patients interpret and adapt to their pain [14]. Well-structured information about the surgery and the whole pathway of care supports patients’ understanding of their physical situation, relieves anxiety and empowers them to actively participate in their recovery [15]. Additionally, managing patients’ expectations prior to surgery is considered important for better physical function post-surgery and satisfaction with surgical outcomes [16, 17]. Long-term success of the joint replacement should be ensured by training the patients how to correctly perform daily activities and movements for gentle use of their artificial joint in daily life [18].

Recently, mobile apps have started to play an important role in monitoring and motivating patients to engage in their health [19]. Mobile Health (mHealth) apps aim to enhance patient involvement and self-management capabilities in patient-centred models of healthcare [20].

There are already a small number of apps that focus on patients undergoing hip or knee replacement [21–24]. Most of them are linked to specific hospitals or surgeons [25] and few of them are offered in German [26]. None of them evaluate the effectiveness on patient outcomes.

The project, pabee (“Patientenbegleiter für endoprothetische Eingriffe” – Patient Companion for Joint Replacement Surgery) wants to combine patient education with the mHealth approach in addition to designing a smartphone based, educative, intervention programme that accompanies patients from the preoperative phase, hospital admission, discharge and all the way until after rehabilitation.

The aim of the project, “pabee” is to develop an innovative mobile app for patients undergoing THR or TKR and to evaluate its efficacy on patient reported outcomes such as physical activity, pain, and quality of life.

The app contains evidence-based content on osteoarthritis and joint replacement procedures in the hip and knee. It aims to address patients’ training needs, support patients’ adherence and self-care competencies and to motivate them to actively participate throughout the entire treatment process.

This paper introduces the app RECOVER-E and details the study protocol of a double-armed controlled trial to determine the impact of the app on patient reported outcomes.

### Study hypothesis

The primary aim of the study is to test the hypothesis that patients undergoing TKR/THR using the app RECOVER-E attain better function in activities of daily living 3 months post-surgery when compared to patients undergoing surgery without using the app (control group).

Further hypotheses assume that patients using the app RECOVER-E experience significantly less symptoms, less pain, better function in sport and recreation, as well as a better knee/hip-related quality of life 3 months post-surgery when compared to the control group. Furthermore, we hypothesize less preoperative anxiety and postoperative pain in patients using the app RECOVER-E when compared to the control group.

## Methods/design

### Design

This study is an unblinded, non-randomized, multi-centre (4 sites), double-arm, controlled trial with 1:1 assignment. The duration of the study is 3 years and includes literature review, development of the app, clinic and patient recruitment, intervention delivery, as well as, data collection and analysis.

### Participants and recruitment

**Fig. 1:**
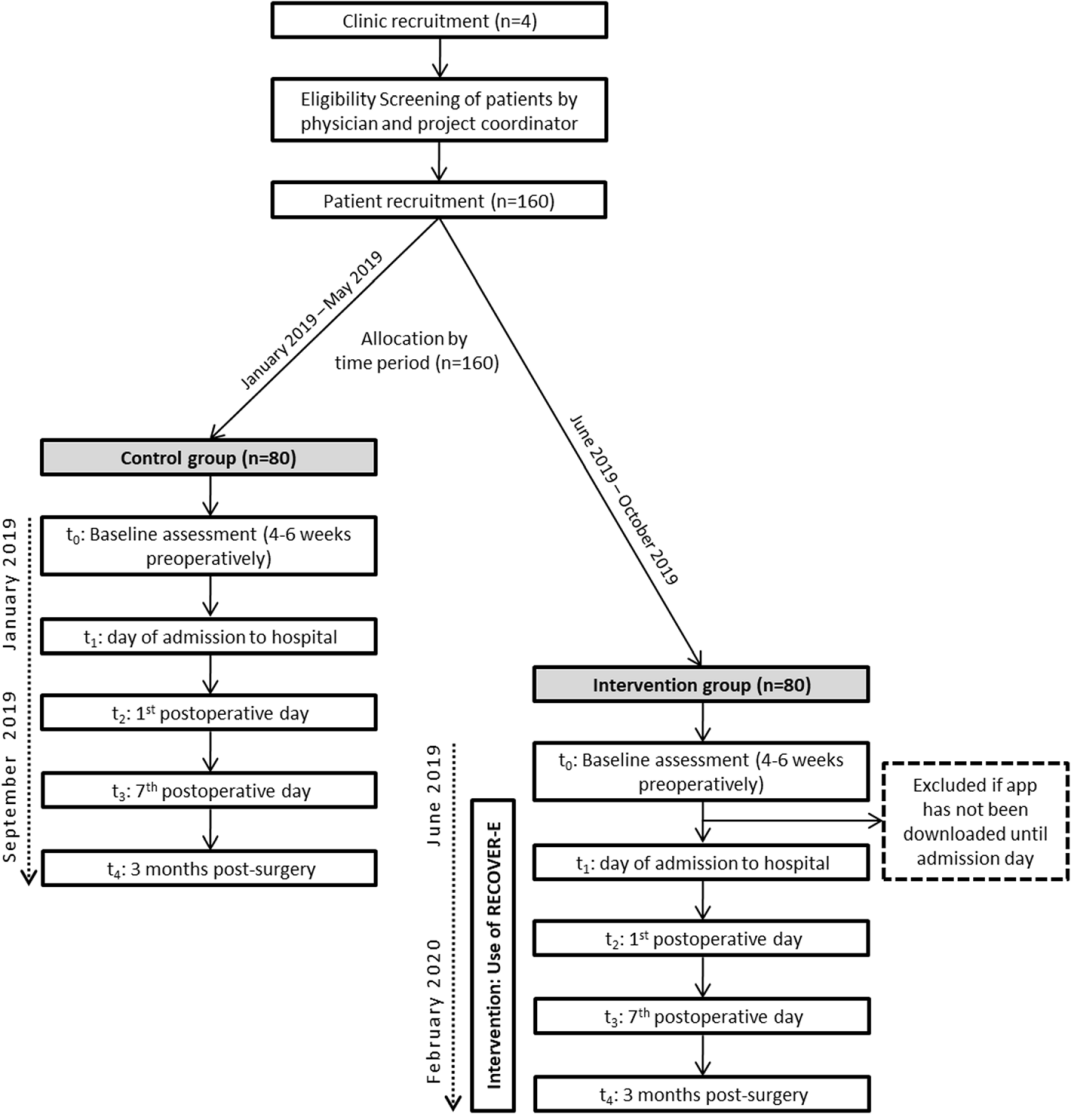
Flow chart of participants in the pabee study

Patients scheduled for THR or TKR will be consecutively recruited in 4 German hospitals in urban and rural areas conducting between 364 and 2300 joint replacement surgeries per year. In addition, the hospitals are publicly, privately and financially associated by the employers’ liability insurance association. The recruitment of the participating patients will be done on initial contact by the inpatient physician and a project coordinator from January 2019 until October 2019 (Fig. 1).

A random assignment of the participants and simultaneous treatment of intervention and control group would carry the risk that people in the control group might learn about the app and its contents from patients of the intervention group. In order to prevent diffusion of treatment effects among participants, patients scheduled for preoperative consultation from January 2019 to May 2019 will be assigned to the control group and patients scheduled for preoperative consultation from June 2019 to October 2019 will be assigned to the intervention group.

### Eligible patients must meet the following inclusion criteria

- scheduled for primary unilateral THR or TKR
- 18 years of age or older
- able to speak and understand German
- Signed informed consent (on paper)
- have a smart phone

### Exclusion criteria

- emergency hip or knee replacement surgery, such as after a fall
- revisions or exchange surgery
- Patients already received a replacement of another joint in the past
- cognitive impairments of all types (as assessed by the attending physician)
- mental illnesses of all types (as assessed by the attending physician)
- Patients, who have not downloaded the app when they were admitted to the hospital (intervention group)
- post-surgical complications, such as infections, allergies, instability, delirium, or other
- limited language skills preventing proper app use or completion of questionnaires

Participants assigned to the intervention group who failed to download or access the app will be excluded. However, project coordinators of the participating hospitals will interview those participants and record their reasons for not engaging with RECOVER-E. These data will be analysed and reported as part of the sample description.

### Control group

The control group will receive standard care as provided by their hospital. This consists of verbal information about the perioperative treatment. Some clinics provide specific folders about the surgery process.

### Intervention group

Patients assigned to the intervention group will, in addition to standard care, receive the smart phone app “RECOVER-E” for iOS and Android devices. Immediately after the baseline data collection (4–6 weeks before surgery, t_0_) participants will receive an access link and instructions on how to download the app on different smartphones. Additionally, they have the possibility to get help from the project coordinators or via the study hotline. Patients register themselves with a personal ID and can immediately start using the app.

### Intervention

Four different stages in the process of knee or hip replacement have shown to be fundamental: the preoperative phase, the intra- and postoperative phase, the rehabilitative phase and the home environment [27].

RECOVER-E accompanies patients throughout the entire treatment process and with integrated gamification playfully encourages them to acquire useful knowledge and engage in exercises and self-management tasks in preparation of their surgery and everyday life after surgery. Provided information and given tasks are adapted to all 4 stages of treatment.

### App development

Considering the possibilities of mobile app design and programming, an iterative development approach of RECOVER-E intended to integrate patient experiences, the expertise of clinicians, and the results of various rapid literature reviews [28].

At an early stage of the app development 3 people over the age of 65 with experience in THR or TKR were interviewed regarding user needs based on their own surgery experience. Important results of these interviews were, for example, discovering that outpatient rehabilitation requires a very early adaptation to the home situation which was apparently difficult without assistance. Physicians, pain nurses and a physiotherapist were consulted specifically in regards to developing appropriate exercises and training recommendations, but also to identify the questions they often experience while treating patients after THR or TKR. To optimize user experience, an expert for app-development was consulted.

Rapid reviews were conducted on the following topics:

- pain management before and after surgery,
- styles, frequencies and potential effects of self-observation and success-tracking of pain, activity or emotional status
- dietary implications of OA and surgery
- motivational features for increase of long-term user engagement

After the first design and functional drafts via Mock-Ups, RECOVER-E had to be streamlined for full process functioning, usability with various use cases in mind, and positive user experience [29].

The prototype of RECOVER-E was tested by conducting a live observation with 4 different users matching the target group. During the whole process of getting the app into operation and manoeuvring through the different options, the user’s view of the smartphone was filmed by a small camera worn around their neck. Users were instructed to read out aloud what appeared on their screen and encouraged to verbally express their first impressions, decisions and thoughts to the interviewer that also sat in the room [30].

Interview transcripts and video-material were analysed by content analysis with a strong focus on usability and the users’ affective reactions and decisions while using the app. The timespan analysed was the first 45–60 min of user experience. Results were used to enhance app functions and design.

### App content

To grant individualised app content, users indicate the joint that will be operated on and their date of surgery at the beginning of the RECOVER-E use. Furthermore, they will be required to specify the date of discharge as well as the beginning and end of their rehabilitation. The content of the app will automatically adjust according to the specific information provided.

The app is comprised of the following features:

- Information and education
- Motivation
- Self-monitoring
- Reminder
- Communication

#### Information and education

Comprehensive text and video material aim to educate the patients and provide information on the disease, the artificial joint, the joint replacement procedure and everyday life after surgery. The preoperative phase focuses on information on the physiology of knee or hip, osteoarthritis, as well as, pain and pain management, but also gives a preview of daily activities with an artificial joint and support possibilities.

Patients will be prepared for the hospital stay and surgery by giving information on how to deal with allergies, general preparation for surgery, medication especially on pain and other pain management interventions [27].

The intra- and postoperative phase contains information on the hospital stay and the procedure itself, complications and how to prevent them, wound care, bowel function and body hygiene.

In the rehabilitative phase and home environment, users are encouraged to take part in their pain management, as well as, in their wound and scar care by frequent monitoring with the tools provided in the app. They are given information on rehabilitation, sports, sleep, sexual activity and returning to work post surgery. Users are offered 8 video clips with short and simple exercises to train muscles and increase flexibility in the legs for either knee or hip surgery (e.g. seated leg extension, elevation of legs while sitting) [31].

RECOVER-E proposes a certain number of repetitions and sets per exercise, which gradually increase after hospital discharge and after rehabilitation to keep training motivationally attractive. However, users are requested to first consult their physiotherapist to individually decide on the appropriate exercise intensity at any stage.

Since emotional well-being is closely and positively linked to the experience of control over one’s own recovery process, RECOVER-E provides important information on symptoms and self-management in the immediate postoperative phase. The app also motivates users to ask family and friends for support in order to bridge difficult and frustrating time periods during the first weeks after surgery [32].

Overall RECOVER-E’s education abilities aim to prepare users for an everyday, joint-sensitive life after their joint replacement surgery.

Text and video material have been developed based on findings of rapid literature reviews and enhanced by expert advice of an orthopaedic surgeon, a physiotherapist, and pain and nursing experts (see App development).

Written and spoken texts use a plain language style and refrain from technical terms or, where absolutely necessary, provide an explanation. To enhance understanding and overall user experience in larger text passages, pictures were selected to elucidate the corresponding subject.

#### Motivation

The motivation of users to a) interact with the app on a regular basis and b) to adhere to app suggestions and tasks is an important aim of the app. As a motivational feature, a daily notification will encourage users to acquire information, engage in exercises according to the treatment stage or congratulates them for special accomplishments. Additionally, the app will comprise gamification by leading the user through various achievement levels resulting in awards.

#### Self-monitoring

Users are constantly encouraged to set and achieve their own goals regarding relevant parameters like pain, physical activity, nutrition and social interaction. They can select from a number of given goals in the “aim section” of the app. These goals were developed according to strict principles of achievability and therapeutic appropriateness and are optional for users.

In the “data collection section” users have the opportunity to enter data for monitoring goal parameters. Goal-setting and self-monitoring widen the users’ reflection on their situation and progress will be visible through graphs in the app throughout all 4 phases.

#### Reminder

Constant continuity of training, frequent self-monitoring of the recovery process and possibilities for self-managing pain are the key elements requiring daily reminders. Medical professionals also see the need to enhance a patient’s self-management ability, particularly when it comes to pain and wound observation to prevent unnecessary readmissions to the hospital [32–34].

One push notification per day reminds users either to do physical exercises, read specific information relevant to the current phase, set new goals or record self-monitoring parameters. The start screen of the app additionally provides icons for pending tasks in the sections “exercise”, “information” and “data collection”. The reminder function is especially important during phases when there is reduced contact with health care professionals, such as before surgery or after the rehabilitation programme. How users experience the effect of reminders on their engagement with the app during the post-hospital phases will be analysed in the qualitative part of the pabee project.

#### Communication

The app comprises an interface for the clinical care teams. This will be used by participating hospitals to provide customized information on centre-specific procedures, teams and other information, the hospitals want to share regarding the organisation of the surgery and hospital stay. Additionally, health data provided by users in their app-based monitoring can be transferred to a specific web site accessible only by clinicians of the participating wards. Users have to give individual permission to transfer information from the pre-hospital and hospital phase only. That information can support the problem-centred conversations with nurses and doctors and give insight on pain- and function-based problems during the preparation phase of the surgery and after the operation.

Another feature to facilitate communication after discharge is an automatic message service to inform the users’ orthopaedist in case of complications after discharge: back at home users are instructed to continue self-assessment of pain, function and to observe their wound. If thresholds are exceeded, the app offers to inform the orthopaedist via fax. Users are free to decide on each individual occasion whether or not that information should be sent to their orthopaedist. They are told to visit a doctor immediately in case they observe a further decline in their health.

### Outcomes

Data presented in Table 1 will be measured in the study.

**Table 1:** Study measures to be collected

Measure	Instrument	t_0_	t_1_	t_2_	t_3_	t_4_
Primary outcome measure						
Activities of daily living (ADL)	KOOS/HOOS subscale ADL (self-reported)	x				x
Secondary outcome measures						
Pain, symptoms, Sport & Rec and quality of life	KOOS/HOOS subscales pain, symptoms, Sport & Rec and quality of life (self-reported)	x				x
Pain at rest	NRS (self-reported)	x	x	x	x	x
Pain during activity	NRS (self-reported)	x	x	x	x	x
Preoperative anxiety and depression	HADS (self-reported)	x	x			
Other measures						
Usage metrics	number of user sessions, length of time participants interact with the app, content they engage with, frequency of recording self-monitoring parameters, number of awards	continuous recording				
Physical activity	IPAQ (self-reported)	x				
Social support	questionnaire (self-reported)					x
Physician visits post-surgery	questionnaire (self-reported)					x
Use of pain medication	medical record, questionnaire (self-reported)		x	x	x	x
Sex, age	medical record				x	
BMI (size, weight)	medical record				x	
Operated joint	medical record				x	
OPS, ICD	medical record				x	
Comorbidities	medical record				x	
Duration of hospital stay	medical record				x	
Type and duration of planned and performed rehabilitation programme	medical record, questionnaire (self-reported)				x	x
Complications post-surgery	medical record, questionnaire (self-reported)			x	x	x
Structural data of hospitals						
- Number of beds - Number of joint replacement surgeries performed over the past 12 months - Number of nurses in the orthopaedic departments: nurses, nursing assistants, Pain Nurses, full-time equivalent - Number of physicians in the orthopaedic departments: total number, number of orthopaedic specialists, full-time equivalent - Process quality management data for the care of patients with THR/TKR (documentation forms and guidelines, interprofessional standard operating procedures for analgesic therapy, pain management guidelines, other pain therapy guidelines, standard operating procedures for patients with THR/TKR, special contracts with other facilities and mandatory health insurance companies for structured patient care) - Regular cooperation with institutions of inpatient or outpatient rehabilitation						

*KOOS* knee injury and osteoarthritis outcome score, *HOOS* hip disability and osteoarthritis outcome score, *NRS* numerical rating scale, *HADS* Hospital Anxiety and Depression Scale, *IPAQ* International Physical Activity Questionnaire, *BMI* body mass index, *OPS* “Operationen- und Prozedurenschlüssel” (= key for surgery procedures), *ICD* International Statistical Classification of Diseases and Related Health Problems, *THR* Total hip replacement, *TKR* Total knee replacement

The primary outcome measure of the study will be the activities of daily living (ADL) subscale of the Knee injury and Osteoarthritis Outcome Score (KOOS) [35] and the Hip disability and Osteoarthritis Outcome Score (HOOS) [36]. The extensive subscale comprises a variety of daily activities (17 items). A 5-point Likert-scale is used and converted into a normalized 100-point score with zero indicating the worst possible function [35–39]. KOOS and HOOS were chosen, because both provide measures of the same subscales, but adapted for knee and hip, respectively. They have been shown to have good reliability and validity in patients undergoing joint replacement [35, 40].

Secondary outcomes are the subscales for pain, symptoms, function in sport and recreation and knee/hip-related quality of life of the HOOS and KOOS, the preoperative anxiety, measured by the Hospital Anxiety and Depression Scale (HADS) [41, 42] as well as their currently experienced pain at rest and pain during activity measured by an 11 point numerical rating scale (NRS; 0 “no pain” – 10 “maximum pain”).

Other measures collected by self-administered questionnaires and further information registered by the medical record include physical activity measured by the International Physical Activity Questionnaire (IPAQ) [43], social support at recovery, post-surgical physician visits, use of pain medication, sex, age, BMI (body mass index; size, weight), operated joint, OPS (“Operationen- und Prozedurenschlüssel” – key for surgery procedures), ICD (International Statistical Classification of Diseases and Related Health Problems), comorbidities, duration of hospital stay in days, type and duration of planned and performed rehabilitation programme and complications post-surgery.

In the intervention group, app use will be monitored throughout the intervention period. This will include the number of user sessions, length of time participants interact with the app, content with which they engage, frequency of recording self-monitoring parameters and number of awards achieved through the gamification system.

For comparability and detailed description of the study sites, structural data of the participating hospitals are also being assessed. Collected data include information on the number of beds, number of joint replacement surgeries performed over the past 12 months, number and full-time equivalents of nurses, nursing assistants, pain resource nurses, physicians and specialists of the orthopaedic department as well as process quality data for the care of patients with THR/TKR and rehabilitation procedures.

### Data collection procedures

Self-reported measures will be collected via self-administered patient questionnaires. Medical data will be transferred from the patients’ medical record into a medical questionnaire by the project coordinators or study nurses.

In total there will be 5 collection points for the patient data presented in Table 1: baseline (4–6 weeks before surgery, t_0_), the day of admission to the hospital (t_1_), the 1st postoperative day (t_2_), the 7th postoperative day (t_3_) and 3 months post-surgery (t_4_).

Patients whose preoperative consultation and recruitment takes place more than 6 weeks before day of surgery will perform the patient survey at baseline (t_0_) via online survey on their own computer at home. Data collection of patients whose preoperative consultation does not exceed 6 weeks before surgery as well as data collection t_1_, t_2_, and t_3_ will be carried out via online survey on tablet PCs in the hospitals provided by the research team.

The follow-up measure (t_4_) will also be an online-based patient survey, for which the patients will receive a link via e-mail as well as a reminder by post 3 months post-surgery. The e-mail address as well as the postal address of participants will be stored in a separate database.

All data of the participating patients will be pseudonymised using ID allocation. Data are accessible to the project team and exported for analysis only after pseudonymisation.

Structural hospital data will be assessed before the collection of patient data by the project team.

### Sample size

Based on the primary outcome ADL subscale of the KOOS/HOOS and the assumable clinical relevant change of 10 points [37, 44, 45], we expect that the group using the mobile app in addition to the standard care will improve at least 10 points more than the control group after having undergone TKR/THR 3 months post-surgery.

To allow for separate analysis of patients with knee and hip OA, 58 knee patients and 58 hip patients are required to detect a difference of 10 points on the HOOS/KOOS ADL subscale (SD 15, power = 0.80 and α = 0.05) [37, 44, 46]. Allowing for 20% dropout [47–49], 5% complications post-surgery [50] and 4% erroneous records, a total of 160 patients will be recruited.

### Data analysis

Baseline data, clinical and demographic characteristics will be presented to show the baseline comparability of the intervention group and the control group. The analysis will include all patients following the intention-to-treat principle, drop-out and loss to follow-up will be described. Data will be checked for completeness and normality. The treatment effect will be evaluated by the change in ADL using between group analyses, such as independent t-test and ANCOVA to adjust for demographic factors such as age, sex, BMI (body mass index), baseline pain, anxiety, joint, comorbidities, physical activity, pain medication, type and duration of rehabilitation and social support on change in outcomes. For comparison within groups paired t-tests will be conducted. Within the intervention group the relationship between usage metrics and patient reported outcomes will be analysed using linear regression analyses.

All statistical analyses will be performed using IBM SPSS Statistics for Windows, version 24 (IBM Corp., Armonk, N.Y., USA).

Results of the study will be presented at conferences and placed in journals.

The controlled trial will be accompanied by multi-perspective qualitative research approaches to understand user decisions and long-term user engagement of the intervention. These studies will be reported elsewhere.

### Ethical considerations

This study is approved by the Ethics Committee of the Medical Association Westphalia-Lippe and of the Faculty of Medicine of the University of Münster (approval number: 2017–329-f-S) and respectively registered via DRKS Data Management (German Clinical Trials Register, ID: DRKS00012744).

Data will be saved, transferred and retained in accordance with European DSGVO (“Datenschutzgrundverordnung” – General Regulation for Data Protection). The analysis will not allow identification of individual participants, and final reports and publications will only consist of aggregated results.

## Discussion

The aim of the study is to enhance patient reported outcomes after surgical hip or knee replacement by using mHealth with evidence-based content and interface.

It seems reasonable to bring patient education to the next level and adapt it to the needs and conditions of modern society. In recent years, the concepts of eHealth (Electronic Health) and mHealth have become increasingly important in patient-centred health care aiming to enhance patient involvement and self-management capabilities [19, 20]. mHealth intends to capitalize on people’s ubiquitous access to mobile phones to provide 24/7 health care [51]. Ideally, it leads to improved health outcomes by increasing patient knowledge, providing social support, enhancing patient-provider communication and saving resources at the same time [52].

However, there is a lack of evidence for the effectiveness of the use of mHealth in patients undergoing joint replacement [53, 54]. This study will investigate the effects of an evidence-based mobile app as an educational intervention on patient reported outcomes of this target group.

Numbers of joint replacements are increasing and shorter hospital stays require solid self-management skills. Evidence supporting the use of mHealth to potentially improve patient outcomes is of particular importance to open up new possibilities for patient care.

A major limitation of the study is the non-randomized assignment of participants. Therefore the comparability of the groups regarding all relevant patient characteristics will be comprehensively analysed.

A second limitation is a potential mode effect due to different survey modes (online based vs. tablet based) at baseline and 3 months post-surgery.

Another limitation and potential source of bias is the exclusion of participants assigned to the intervention group, but who failed to download or access the app. We have chosen to do this because those participants miss out on engaging with app contents for the preoperative phase that are expected to have an effect on primary and secondary outcomes. We will, however, analyse and report patients’ reasons for not engaging and consider this aspect regarding the generalisability of our findings.

Design and structure of the app were specifically developed to meet the needs of an older target group. However, a lack of patient technology literacy cannot be entirely eliminated, thereby, as a further limitation, negatively affect patient adaptation and compliance to mobile intervention.

### Trial status

At the time of submission of the manuscript in May 2019, recruitment of the patients for the control group is ongoing. App development is in the final testing phase.

## Data Availability

Not applicable.

## Abbreviations

ADL: Activities of daily living
DSGVO: “Datenschutzgrundverordnung” – General Regulation for Data Protection
eHealth: Electronic Health
ERP: Enhanced recovery programmes
HADS: Hospital Anxiety and Depression Scale
HOOS: Hip disability and osteoarthritis outcome score
ICD: International Statistical Classification of Diseases and Related Health Problems
IPAQ: International Physical Activity Questionnaire
KOOS: Knee injury and osteoarthritis outcome score
mHealth: Mobile Health
NRS: Numerical rating scale
OPS: “Operationen- und Prozedurenschlüssel” – key for surgery procedures
THR: Total hip replacement
TKR: Total knee replacement
